# Exome Sequencing of Native Populations From the Amazon Reveals Patterns on the Peopling of South America

**DOI:** 10.3389/fgene.2020.548507

**Published:** 2020-10-29

**Authors:** André M. Ribeiro-dos-Santos, Amanda Ferreira Vidal, Tatiana Vinasco-Sandoval, João Guerreiro, Sidney Santos, Ândrea Ribeiro-dos-Santos, Sandro J. de Souza

**Affiliations:** ^1^Genetics and Molecular Biology Graduate Program, Instituto de Ciências Biológicas, UFPA, Belém, Brazil; ^2^Oncology and Medical Science Graduate Program, Núcleo de Pesquisas em Oncologica, UFPA, Belém, Brazil; ^3^Instituto do Cérebro, UFRN, Natal, Brazil; ^4^Bioinformatics Multidisciplinary Environment (BioME), Instituto Metrópole Digital, UFRN, Natal, Brazil; ^5^Institute of Systems Genetics, West China Hospital, Sichuan University, Chengdu, China

**Keywords:** Amazon, native populations, exome, genomics, SNPs (single nucleotide polymorphism)

## Abstract

Studies on the peopling of South America have been limited by the paucity of sequence data from Native Americans, especially from the east part of the Amazon region. Here, we investigate the whole exome variation from 58 Native American individuals (eight different populations) from the Amazon region and draw insights into the peopling of South America. By using the sequence data generated here together with data from the public domain, we confirmed a strong genetic distinction between Andean and Amazonian populations. By testing distinct demographic models, our analysis supports a scenario of South America occupation that involves migrations along the Pacific and Atlantic coasts. Occupation of the southeast part of South America would involve migrations from the north, rather than from the west of the continent.

## Introduction

The peopling of the Americas remains a fascinating, still controversial, and topic. While there has being significant advances in the last decade mainly due to genomics approaches being used in contemporaneous and ancient samples, critical issues remain unanswered, and especially regarding South America.

It is now widely accepted that Native American founders moved from East Asia through Beringia, a land bridge between Northeast Asia and the extreme Northwestern America, and rapidly populated the whole continent (Feathers et al., 2010; Dillehay et al., 2015). Archeological evidences on the American side of Beringia suggested a migration around 16,000 years ago (16 kya). Genetic differences between Native Americans and East Asians, however, indicate an older split between the two groups, more around 23 kya (Raghavan et al., 2015; Llamas et al., 2016), which led to the suggestion that the American founders stayed in Beringia for few 1,000 years (Tamm et al., 2007; Fagundes et al., 2008). More recent evidence from archeological, linguistic and genetic data suggest at least three major migratory routes from Beringia to the continent (Reich et al., 2012; Raghavan et al., 2015). The major route south was a Pacific coastal one with several evidences suggesting an extremely rapid occupation of the whole west coast of the continent. First, solid archeological evidences in the south of Chile showed that humans reached that point around 14 kya (Dillehay et al., 2015). Furthermore, genetic divergence between Central and South American populations indicate a split around 13 kya (Gravel et al., 2013).

The peopling of South America is, however, more obscure. While is clear that a Pacific coastal route was rapidly used to reach the extreme south, it is generally believed that there was also an Atlantic coastal route toward the east (Wang et al., 2007; Bodner et al., 2012; Reich et al., 2012; Gómez-Carballa et al., 2018). How the interior part of South America, including the Amazon region, was occupied by our ancestors is a matter of debate, with several possible migratory routes from both west and north (Rothhammer and Dillehay, 2009).

To make the scenario even more complex, recent reports (Raghavan et al., 2015; Skoglund et al., 2015) have identified an Australasian signal in genomic data from some groups of Native Brazilians (as well as from an ancient individual from the northern part of China). The original population that putatively contributed to this Australasian signal was named “Population Y”. More recently, Posth et al. (2018) and Moreno-Mayar et al. (2018) sequenced several ancient American individuals, including ones from Lagoa Santa in the Southeast part of Brazil, and found conflicting results. While Moreno-Mayar et al. (2018) found evidence for an Australasian genetic signal, Posth et al. (2018) failed to find such signal in different fossils samples separated geographically by few dozen kilometers. Both papers, however, emphasize the complex and dynamic migratory landscape from North to South America.

Since there is an extensive admixture in the American population due to European colonization and African slave trade, uniparental genetic systems (especially mitochondrial DNA) have been used to reconstruct Native American genetic origin (Bodner et al., 2012; Roewer et al., 2013; Gómez-Carballa et al., 2018). More recently, access to isolated Native Americans, with no or limited admixture with other ethnic groups, have contributed significantly toward the definition of the genetic landscape of the first Americans (Reich et al., 2012; Crawford et al., 2017; Harris et al., 2018). While there are genetic data from several populations across the Pacific coast and Andes in South America, there is a shortage of data from Native Brazilians, especially from the east part of the Amazon basin.

To contribute to the reconstruction of the genetic history of Native Brazilian populations and address issues related to the peopling of South America, we analyzed exome data from 58 individuals from 8 different tribes scattered through the east of the Amazonian region. We have also included in our analysis genomic data from several other projects covering extant and fossil samples from a broad geographic extension, covering North, Central and South America regions. A marginal Australasian signal was found in two of the Amazonian populations sequenced here: Araweté and Zo’é. We confirmed that there is a clear genetic distinction between populations from the east and west of South America. This comparative analysis also allowed us to model the demographic movements in the early occupation of South America and suggest that the southeast part of the continent was occupied mainly by migrations from the Amazonian region.

## Results

### Exome Sequencing of 58 Native Brazilians From 8 Amazonian Populations

**FIGURE 1:**
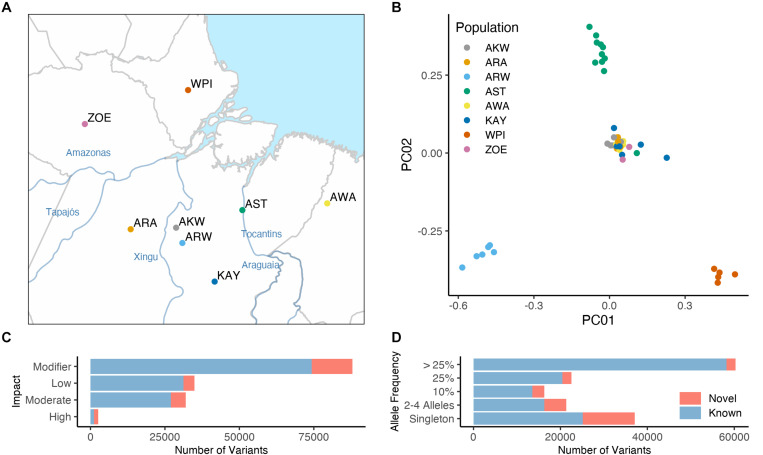
Exome sequencing results of 58 Native Brazilians from eight distinct populations. The Native American populations investigated here are identified as follow: Arara/Arara do Iriri (ARA); Asurini do Koatinemo (AKW); Asurini do Trocará (AST); Araweté (ARW); Kayapó/Xikrin (KAY); Zo’é (ZOE); Wayãpy (WPI); and Awa-Guajá (AWA). **(A)** Indicate those populations geographic location the Amazon eastern basin. **(B)** PCA including only Native Brazilians sequenced here. **(C,D)** Number of known (identified by 1000 Genomes Project, ExAC, or gnomAD) and novel variants in our dataset classified by variant impact **(C)** and allele frequency **(D)**.

We sequenced the exome of 58 Native Brazilians samples from eight populations located at the east part of the Amazon basin (Figure 1A). These included Araweté (ARW), Zo’é (ZOE), Wayãpy (WPI), and Awa-Guajá (AWA) from the Tupi-Guarani language group; Asurini do Koatinemo (AKW), and Asurini do Trocará (AST) from the Asurini language group, which belong to the Tupi-Guarani language truck; Arara/Arara do Iriri (ARA) from the Karib language group; and Kayapó/Xikrin (KAY) from the Macro-Jê language group. In terms of geographical location, ARA, AKW, and ARW are located on the Xingu River basin; KAY is located in the southeastern region of the Pará state; AST is located near the basin of the Tocantins river; ZOE inhabit a region between Cuminapanema and Erepecuru rivers in the northwestern portion of the Pará state; WPI inhabits the Oiapoque region in the north of the Amazon basin; and AWA, who are the last nomadic population in Brazil, they inhabit a vast region of the Maranhão state.

Variant calling in the exome data identified 132,794 single nucleotide variations (SNVs) and 14,102 indels. Supplementary Table 1 lists all SNVs and INDELs in all individuals with their corresponding putative impact as defined by SnpEff (Cingolani et al., 2012) and the proportions of mutations by type of impact are shown in Figure 1C. A significant fraction of all variations is specific to the populations sequenced here (Figures 1C,D). Principal component analysis (PCA) of the sequenced individuals is shown in Figure 1B (expanded in Supplementary Figure 1) and indicates the separation between the different populations. When compared to worldwide samples, all individuals sequenced here clustered with other Native American populations on a PCA (Supplementary Figure 2). An additional run of homozygosity (RoH) analysis clearly show that the populations sequenced here have a similar pattern of RoH to other native Brazilians populations, like Surui and Karitiana (Supplementary Figure 3).

### Comparisons With Other Extant and Ancient Populations

**FIGURE 2:**
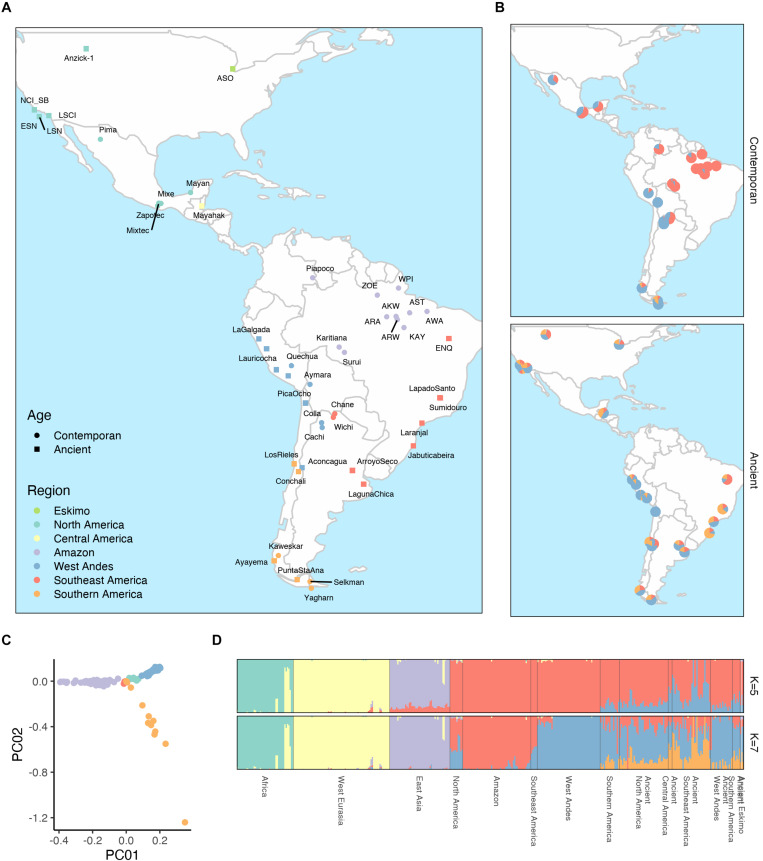
Geographic and genetic structure of Native South American populations. **(A)** Indicates the geographic location of all contemporaneous and ancient populations investigated here colored according to their geographic region into Eskimo, North America, Central America, and the South America was further divided into Amazon, West Andes, Southeast America and Southern America. **(B)** illustrates the average contribution of putative ancestral components among contemporaneous and ancestral populations inferred by an unsupervised ADMIXTURE analysis with seven ancestral components (*K* = 7). **(C)** PCA analysis of all contemporaneous Native American samples investigated here. **(D)** This represents estimates of ancestral contribution estimated by unsupervised ADMIXTURE analysis with five (*K* = 5) and seven (*K* = 7) where each ancestral component is color-coded and the indivual ancestry is presented as vertical bars.

The genetic structure of Native Brazilian populations was explored and compared to other worldwide populations, with emphasis to Native American populations, including contemporaneous and ancient individuals (see methods and Supplementary Table 2). Figure 2A shows the geographic distribution of all Native American populations included in our analysis. The PCA analysis of all contemporaneous Native American samples (Figure 2C and Supplementary Figure 4) showed that they clustered according to their geographic regions, as indicated in Figure 2A.

When running an unsupervised ADMIXTURE (Alexander et al., 2009) analysis, we found a clear Native American and Ancient Native American genetic component with five ancestral components (*K* = 5), the other three components being African, European and East Asian (Figure 2D and Supplementary Figure 5). The Native American component further splits into a West Andean and Amazonian on the model with seven ancestral components (*K* = 7; Figure 2D and Supplementary Figure 5) and Figure 2B illustrates the average distribution of these components per population among contemporaneous and ancient samples. Both models with five and seven ancestral components best fit the data according to cross-validation estimates (Supplementary Figure 6). The same overall patterns among Native Americans were observed using TreeMix (Pickrell and Pritchard, 2012) with a clear distinction between samples from the Amazon, West Andes and South regions (Supplementary Figure 7). Samples that presented a non-Native American contribution higher than 10% in the analysis with five ancestral components were considered outliers and excluded from all other analyses.

**FIGURE 3:**
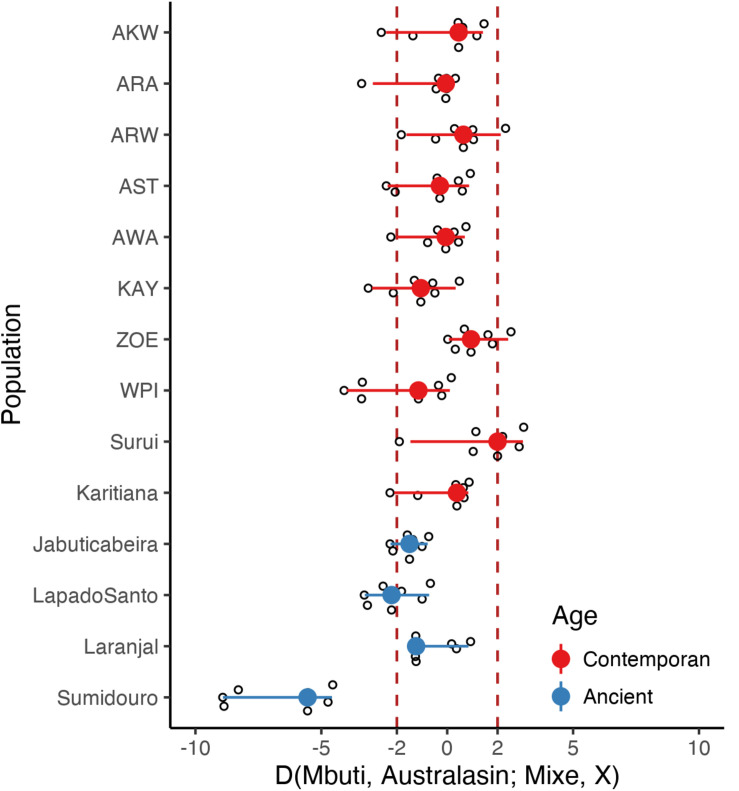
Investigation of an Australasian component among Native Brazilians. To investigated the hypothesis of an Australasian signal among Native South America populations, we computed *D*(Mbuti, Australasian; Mixe, *X*) *Z*-value where X represents the test population (indicated in the *y*-axis) and Australasian represents Andaman, Australian, Papuan New Guinea, Bougainville, Dusun, Igorot, or Maori. *Z*-values greater than three (represented by the dashed vertical lines) indicate an Australasian signal. The point range indicates the *D*-statistic *Z*-value median and 95% interquartile range among all Australasian populations and were colored according to the test population age.

Since there are controversial evidences of an Australasian genetic signal in South America, we decided to test for that gene flow signal in the Amazonian populations studied here. Thus, we computed *D*-statistics of the form *D*(Mbuti, Australasian; Mixe, *X*), where X represents each one of the fourteen Brazilian populations (sequenced here or obtained from the public domain) and Australasian indicate one of the following populations: Andaman, Australian, Papuan New Guinea, Bougainville, Dusun, Igorot, and Maori (Figure 3). While we confirmed an Australasian signal in Surui, we found only a marginal signal in two other Amazonian populations: Araweté and Zo’é, although no statistical significance was reached. Furthermore, we were unable to confirm the Australasian component in ancient samples from Sumidouro as reported by Moreno-Mayar et al. (2018). Actually, samples from Sumidouro presented a strong negative Z-score for the above *D*-statistics, probably reflecting their unique genetic structure.

### Formal Tests for Admixture Between Native American Populations

Based on the assumption that the occupation of South America started by at least two different coastal routes, a Pacific and an Atlantic one, we decided to further test hypothetical demographic models with the following *D*-statistic, where X represents the test population; Amazon, West Andes and Southern America represents any population from these regions; and Mbuti was used as outgroup:

(A) *D*(Mbuti, X; Amazon, Southern America), which test the gene flow from Amazon to population X in regard to southern populations.
(B) *D*(Mbuti, X; Amazon, West Andes), which tests the gene flow between Amazon and West Andes to population X.
(C) *D*(Mbuti, X; Southern America, West Andes), which test the gene flow from West Andes to population X in regard to southern populations.

**FIGURE 4:**
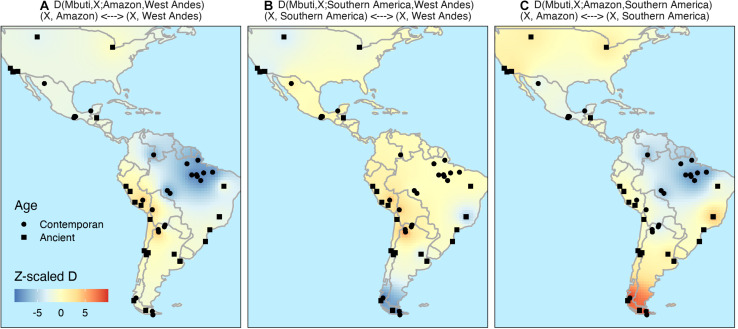
Inverse distance weighting (IDW) interpolation of *D*-statistic to formally test migration models. We explore different demographic models of migration by computing the outgroup *D*-statistic as *D*(Mbuti, *X, Y*, and *Z*) Z-score where *X* is the test population, *Y* and *Z* are the populations which the genetic drift is being compared. Where *Z*-scores higher than three (red) indicated a closer relation of *X* to *Z* and lower than –3 (blue) a closer relation of *X* to *Y*. Here we tested the following: **(A)** *D*(Mbuti, *X*; Amazon, West Andes); **(B)** *D*(Mbuti, *X*; Southern America, West Andes); and **(C)** *D*(Mbuti, *X*; Amazon, Southern America).

As expected, Figures 4A,C confirms that all populations sequenced here have a clear Amazonian signal since a significative negative signal indicate a closer relation of X to Amazon in both maps. The data also supports the view mentioned above about the genetic distinction between western and eastern South American Native populations as demonstrated in the test *D*(Mbuti, X; Amazon, West Andes) (Figure 4A). The same test shows also a stronger genetic similarity between Amazonian and Southeast America populations when compared to Andean populations. Finally, Figure 4B presents evidence of gene flow between samples from Lagoa Santa (Brazil) and ancient samples from the west part of North America (as has been shown by Posth et al., 2018).

### Further Tests on Different Demographic Models

We can envision few demographic scenarios for the occupation of central parts of South America, including the Amazonian region. Four possible scenarios will be discussed: (1) groups from the Atlantic route eventually turned south to occupy the Amazon basin and the Southeast of the continent; (2) groups from the Pacific route eventually turned east and occupied the central parts of the continent (likely in different waves from north to south); (3) Andean groups in the south migrate east and eventually turned north to occupy the south/central part of Brazil and the Amazonian region; and (4) a mixed model involving at least two of the models above. Since scenario 4 above is difficult to test with the present limitation in sample size, this leaves us with scenarios 1–3.

We modeled these scenarios as admixture graphs (Supplementary Figure 8) and computed the models likelihood and worst *f*_4_ statistic using qpGraph from admixtools v.2.0 (available at ^1^) while varying the population representing each leaf nodes. Overall model 1 better fitted the data with smaller likelihood scores and *f*_4_ statistics with few tests presenting the worst absolute *f*_4_ below 3 (Supplementary Figures 9, 10).

## Discussion

The exome sequencing of 58 Native Americans from the east part of the Amazonian region contributed to clearly define an Amazonian genetic signature. All the sequenced individual clustered with Karitiana, Surui and Piapoco and were distant from Andean populations (Figure 2C). A genetic distinction between western and eastern South America populations had already been noticed by others (Tarazona-Santos et al., 2001; Wang et al., 2007; Reich et al., 2012; Barbieri et al., 2019; Gnecchi-Ruscone et al., 2019). Data from the analyses reported here also suggest that populations from the southeast of Brazil and north of Argentina are more similar in their genetic structure to Amazonian populations than to Andean populations. This would suggest that the occupation of the central part of South America involved a migratory route from the north of Brazil, rather than an occupation from the west.

Overall, we tested three different demographic models: (1) groups from the Atlantic route eventually turned south to occupy the Amazon basin and the Southeast of the continent; (2) groups from the Pacific route eventually turned east and occupied the central parts of the continent (likely in different waves from north to south); and (3) Andean groups in the south migrate east and eventually turned north to occupy the south/central part of Brazil and the Amazonian region. The clear genetic distinction between western and eastern populations in South America, seen by others (Tarazona-Santos et al., 2001; Wang et al., 2007; Reich et al., 2012; Barbieri et al., 2019; Gnecchi-Ruscone et al., 2019) and confirmed here, weakens scenario 2, since we would expect a gradual transition of the Andean genetic signature from west to east. Although this may have occurred in the Peruvian Amazon, as shown by Harris et al. (2018) and Gnecchi-Ruscone et al. (2019), this does not seem to be true for most of eastern populations, even Kariatiana and Surui (Figures 2B,C) that are geographically much closer to the Andes than the other populations sequenced here.

Since native populations in the southeast (southeast of Brazil and/or north of Argentina) are closer to the native populations in the Amazonian region than to Andean populations, as observed by many authors (Reich et al., 2012; Gómez-Carballa et al., 2018; Harris et al., 2018) and here (Figures 2, 4), we decided to test whether we could detect significant gene flow between Andean and southeast populations. The absence of such signal would give more support to the migration scenario 1, as discussed by Gómez-Carballa et al. (2018), who found only one population in the south-east (the Diaguitas) with a certain level of admixture with the Andean populations. Data in Figure 4 gives strong support to such scenario with only Wichi showing significant admixture with Andean populations. They are located at the east side of the Andes and north of Argentina, and that signal may due to recent admixture with West Andes populations. We have also used qpGraph to test the three demographic models above.

All these results give support to scenario 1, in which the southeast part of continent was populated from the north, rather than the west. Wang et al. (2007) had already proposed that native groups from the central/south of Brazil (more specifically Ache, Guarani and Kaingong) came from the north through an Atlantic coastal route. More recently, Gómez-Carballa et al. (2018), based in mitochondrial and autosomal variations, suggested that gene flow between populations that followed the Pacific and Atlantic routes were extremely limited and the Atlantic route was the major source for the peopling of the southeast part of South America.

Due to the controversy regarding a possible Australasian genetic signal in Native Americans and ancient samples from South America (Moreno-Mayar et al., 2018; Posth et al., 2018), we decided to test for the presence of that putative signal in all 58 samples sequenced here. A trend was observed in two populations: Zo’é and Araweté, with both signals not reaching statistical significance. The scattered geographic pattern, together with the marginal strength of this Australasian signal in the samples in which it was detected, raises the possibility of an artifact. More samples are needed before one can reach a conclusion.

The present study explored genetic data of Native Americans through whole-exome sequencing and investigated the history of occupation and expansion of these populations in South America. Our data support an occupation model with separate migration waves, most likely through a Pacific and Atlantic route with the southeast part of the continent being occupied by migrations from the Amazonian region.

## Materials and Methods

### Ethical Disclaimer

The samples were collected from adult individuals (between 18 and 50 years old) from eight Native American populations residents of the Brazilian Amazon. They were collected as part of two projects developed by the Laboratório de Genética Humana e Médica (LGHM) and approved by Brazilian National Committee on Research Ethics – CONEP (identified by N^*o*^ 1062/2006 and 123/98). All participants signed a free-informed consent as well as the tribe leaders and when necessary a translator explained the project and the importance of the research. Their materials were collected according to the Declaration of Helsinki.

### Exome Library Preparation

The peripheral blood of the subjects was collected into vacutainer tube with EDTA. DNA was extracted using the phenol-chloroform method (Sambrook and Russell, 2006), quantified using Nanodrop fluorometer (Thermo Fisher) and integrity evaluated by electrophoresis in 2% agarose gel.

Whole-exome sequencing libraries were prepared using Nextera Rapid Capture Exome (Illumina) and SureSelect Human XT all exon V6 (Agilent) kits following the manufacturer recommendations. The libraries were sequenced in the NextSeq 550 sequencing platform (Illumina) in 4 NextSeq 500/550 High Output Kit runs with approximately 16 samples each.

### Read Processing and Variant Calling

Sequencing reads were trimmed for Illumina adaptors and filtered using Trimmomatic v.0.36 (Bolger et al., 2014) and the remaining reads were aligned to the human reference genome hg19 (available at ^2^) using BWA MEM v0.7 (Li, 2013; Li and Durbin, 2009). PCR duplicates were removed using Samblaster v.0.1 (Faust and Hall, 2014) and the mapped reads were sorted and indexed using Samtools v1.8 (Li et al., 2009) and Sambamba v.0.6 (Tarasov et al., 2015). Finally, the mapped bases quality score was recalibrated with GATK v.4.0.0 BaseRecalibrator and ApplyBQSR walkers. Supplementary Table 3 includes a detailed table of our samples QC metrics. The code use to process all samples is available at https://github.com/andremrsantos/exomeseq-nf.

Two strategies were applied for variant calling, an unguided and a guided approach. The unguided approach aimed to identify potential new variants and is consistent with the GATK best practice recommendations (Van der Auwera et al., 2013). Variants within the targeted regions were identified using GATK v4.0.0 HaplotypeCaller and called as a cohort with GenotypeGVCF. SNVs quality was calculated measured based on known variants from the GATK resource bundle, which included variant datasets such as 1000 Genomes Project (The 1000 Genomes Project Consortium et al., 2015) and HapMap high confidence. The resulting variant file was annotated using SnpEff v.4.3 (Cingolani et al., 2012) and vcfanno v.0.3 (Pedersen et al., 2016) to include the variant effect, clinical importance according to ClinVar (Landrum et al., 2016), and GWAS annotations from GWAS catalog (MacArthur et al., 2017), allele frequency from ExAC, gnomAD (Lek et al., 2016) and 1000 Genomes Project (The 1000 Genomes Project Consortium et al., 2015).

The guided approach aimed to maximize the number of comparable sites for population analysis. First, we selected all biallelic SNV from the Simons Genome Diversity Project or SGDP (Mallick et al., 2016) within the union of all targeted regions and genotype those variants using HaplotypeCaller in the GENOTYPE_GIVEN_ALLELES mode for all samples included, except those from Pagani et al. (2016), which did not raw sequencing data available, and the SGDP since they were already available. The individual VCFs were aggregated using plink v.1.9 (Chang et al., 2015) excluding variants with overall missing genotype rate above 25%. Further excluded samples inferred as full siblings by KING v.2.1.4 (Manichaikul et al., 2010) and those with less than 90% contribution of either Native American or Ancient Native American contribution when conducted an unsupervised ADMIXTURE (Alexander et al., 2009) analysis with five putative ancestral components.

### Modern and Ancient Samples Collection

To conduct the population analysis we have included present-day worldwide human data from Simons Genome Diversity Project (Mallick et al., 2016) public dataset, which included high-coverage genome sequencing of 10 Native American populations. We’ve also included other Native American population data from Raghavan et al. (2015), Crawford et al. (2017), Pagani et al. (2016), and de la Fuente et al. (2018), and ancient samples from Rasmussen et al. (2014), Scheib et al. (2018), Moreno-Mayar et al. (2018), and Posth et al. (2018). A full table of the samples included is available in Supplementary Table 2. The final dataset included 980.592 variants and 421 samples, including 132 contemporary and 97 ancient Native American samples after all filters.

### Population Structure, f and D-Statistic Analysis

We broadly investigate the genetic structure between all samples using an unsupervised ADMIXTURE v.1.3 (Alexander et al., 2009) clustering analysis to infer contribution of putative ancestral components and a principal component analysis using flashPCA v.2.0 (Abraham et al., 2017). FlashPCA was ran using the default options and ADMIXTURE was ran with 5 cross-validation iterations and varying the number of putative ancestral components (*K*) between 2 and 10. To improve FlashPCA performance, rare variants (allele frequency bellow 1%) were excluded since they have a limited contribution to the analysis.

We also investigated the relationship between the investigated populations running TreeMix v.1.13 (Pickrell and Pritchard, 2012) including up to five migration edges, blocking variants to reach approximately 20.000 blocks and measuring branches’ confidence by 500 bootstrap iterations. Further explored our samples distribution of runs of homozygosity (ROHs) identified using plink v1.9 (Chang et al., 2015) and compared to other worldwide and Native American samples.

The unbiased gene-flow f_3_ and D-statistic were computed according to Reich et al. (2009) and Patterson et al. (2012), respectively. These metrics were used to evaluate various gene-flow models among the Native American populations. The standard error and Z-score of these statistics were estimated through a weighted block jackknife approach as suggested in Reich et al. (2009) and Patterson et al. (2012) and similar to the one implement in TreeMix v.1.13 (Pickrell and Pritchard, 2012). Briefly, the statistics were measured in genomic blocks of approximately 100 variants which were weighted according to the number of sites and used to compute the statistic mean, standard error and Z-score. Models of migration were also explored by fitting our data to admixture graphs using qpGraph from Admixtools v2.0 (available at ^3^).

A detailed description of all analysis conducted here is included to Supplementary Material 1, the code used to produce all figures and conduct all analysis implemented here is available in the companion repository available at https://github.com/andremrsantos/paper-sa-population.

## Data Availability Statement

The data obtained from the public domain are available at the European Nucleotide Archive (ENA https://www.ebi.ac.uk/ena) under the accession numbers: PRJEB9586, PRJNA393593, PRJEB24629, PRJNA229448, PRJEB25445, PRJEB29074, PRJEB28961, and PRJEB12437 and the sequencing data generated here are available at the ENA database under the accession number PRJEB35045.

## Ethics Statement

The study was review and reviewed and approved by Brazilian National Committee on Research Ethics – CONEP (identified by Nos. 1062/2006 and 123/98). All participants signed a free-informed consent as well as the tribe leaders and when necessary a translator explained the project and the importance of the research.

## Author Contributions

SdS, ÂR, and SS conceived the study. JG was responsible for collecting all samples. AMR was responsible for all bioinformatics analysis. AV and TV-S were responsible for sample processing and sequencing. SdS and AMR wrote the manuscript. All authors made contributions to the text and approved the final version.

## Conflict of Interest

We would like to mention that SdS is co-founder of DUNA Bioinformatics. The remaining authors declare that the research was conducted in the absence of any commercial or financial relationships that could be construed as a potential conflict of interest.

## Footnotes

1 https://uqrmaie1.github.io/admixtools/index.html

2 http://genome.ucsc.edu

3 https://uqrmaie1.github.io/admixtools

## Supplementary Material

Supplementary Figure 1 PCA overview of this study Amazon Native American samples. Results of the PCA analysis conducted among only the samples sequenced in this study. Each of the eight populations investigated are indicated by the points color. **(A)** 2D Scatter plot representation of all first four principal components combinations. **(B)** Barplot representation of the variance explained by each principal component.

Supplementary Figure 2 PCA overview of all present-day worldwide sample analysis. Results of the PCA analysis conducted among all present- day worldwide samples included in this study. The samples were colored according to their geographic region with AFR indicating African populations, EAS East Asian, EUR European, OCE Oceanian and NAT Native American. **(A)** 2D Scatter plot representation of all first four principal components combinations. **(B)** Barplot representation of the variance explained by each principal component.

Supplementary Figure 3 Run of Homozygosity distribution in African, European, East Asian and Native American populations by study. The runs of homozygosity (ROH) were identified using plink v.1.9 and their distribution representation are colored according to the population geographic region and further distinguished by sample source study as indicated by the point shape. **(A)** Presents pointrange plots representing the median and 95% data interval of the total ROH length within each ROH length category. **(B)** Is a scatter plot showing the relation of total ROH length and the number of ROH of each individual. **(C)** Presents a pointrange plot representing the median and 95% data interval of total ROH length for each contemporan population included in the analysis.

Supplementary Figure 4 PCA overview of all present-day Native American samples. Results of the PCA analysis conducted among all present-day native american samples included in this study. The samples were colored according to their geographic region, where North America was kept as is and South America was divided into: Amazon, West Andes, Southeast America and Southern America. **(A)** 2D Scatter plot representation of all first four principal components combinations. **(B)** Barplot representation of the variance explained by each principal component.

Supplementary Figure 5 Shared ancestry and genetic structure of the dataset. An unsupervised clustering analysis was performed with ADMIXTURE varying the number of putative ancestral components (*K*) from 2 to 10. Each horizontal bar represents an individual in the dataset and the colors represent their ancestry components assignments for each *K* putative ancestral. The samples were grouped according to their populations and geographic region which are indicated in *y*-axis and delimited by horizontal black bars and panel boxes, respectively.

Supplementary Figure 6 ADMIXTURE genetic structure models likelihood and error. An unsupervised clustering was performed with ADMIXTURE to infer the contribution of putative ancestral components (*K*) varying from 2 to 10. Each model likelihood and cross-validation (CV) error were measured and presented above as a dot line plot. The measures indicate that the model with *K* = 7 presented the best fit to the data, considering it presented the lowest CV error.

Supplementary Figure 7 Maximum Likelihood trees and admixture graph inferred. The maximum likelihood tree and admixture graphs were inferred with Treemix allowing up to five migration events between the branches. For this analysis the variants were blocked into 20,000 blocks and branch support were measured by 500 bootstrap iterations. The branches are colored according to the most recent common ancestor (MRCA) region and their support are indicated in both trees for those with over 75% support. Migration branches are indicated as curves and colored according to their migration ratio. In **(A)** we depict the maximum-likelihood tree produced by Treemix and in **(B)** we show the consensus tree produced by the bootstrap iterations where we merged all branches with less than 75% support.

Supplementary Figure 8 Admixture graph representation of migration models proposed. To evaluate the migration models proposed in the manuscript, we’ve implemented them as admixture graphs to evaluate using qpGraph from ADMIXTOOLS. Admixture edges are represented as dashed lines and the four main South America regions and outgroup are indicated by their respective colors. Model 1 proposed that after being isolated from South Native American populations, West Andes and Amazon populations separated and the current South East is a product of their admixture. Model 2 instead proposed that Southeast America is a product of Southern America and Amazon admixture and Model 3 proposed that Southeast America shares a common ancestor to Amazon populations.

Supplementary Figure 9 Assessment of top ten fitted admixture graphs. The admixture graph for all three models were fitted using qpGraph from ADMIXTOOLS, varying the sample node representing each region. Here we present the top eight admixture graphs with the lowest likelihood. Admixture edges are represented by dashed lines with their contribution indicated as well as the edges Fst.

Supplementary Figure 10 Assessment of fitted admixture graphs. Each migration model proposed were evaluated with qpGraph function from the Admixtools2 package varying samples representing each leaf node (Out, Southern America, Amazon, West Andes, and Southeast America). Here we present a scatter plot of each model likelihood score (where smaller scores are better) and worst F4 obtained with either Mbuti, Han, Mayan, or Pima outgroups. Overall model 1 presents consistently better results than all others. The black pointrange indicates median and 95% data interval likelihood score and worst F4 for each model and outgroup.

Supplementary Table 1 SNV and INDELs identified by the Native American whole-exome sequencing generated here.

Supplementary Table 2 Sample and population data for those included in this study.

Supplementary Table 3 Sequencing quality control metrics for the samples generate here.

Supplementary Material 1 Detailed Methods.
