# Supplementary figures and images for: Exome Sequencing of Native Populations From the Amazon Reveals Patterns on the Peopling of South America
Source: Front Genet. 2020 Oct 29;11:548507. doi: 10.3389/fgene.2020.548507 (PMC7660019; doi:10.3389/fgene.2020.548507)

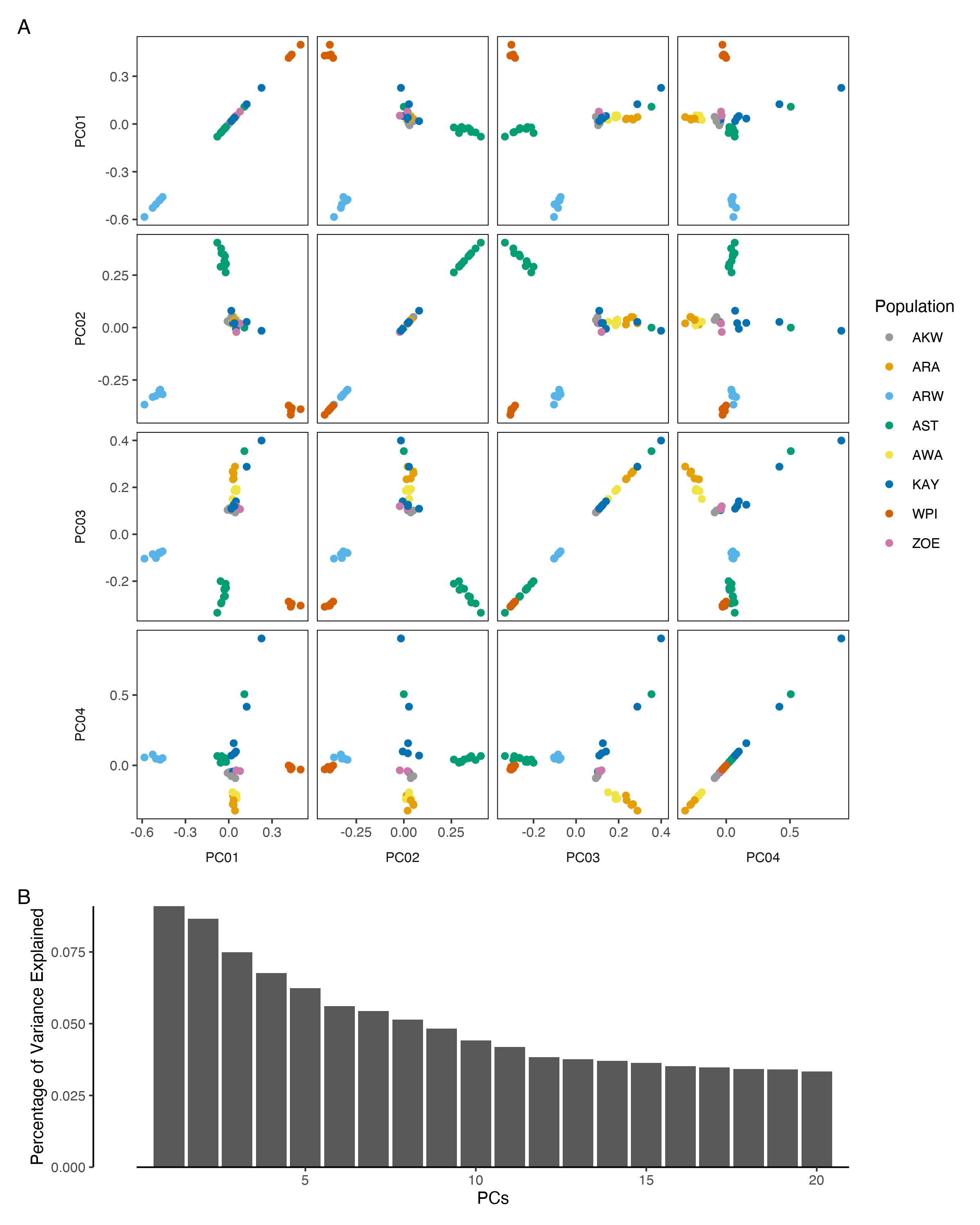

Supplement: Supplementary Figure 1 — PCA overview of this study Amazon Native American samples. Results of the PCA analysis conducted among only the samples sequenced in this study. Each of the eight populations investigated are indicated by the points color. (A) 2D Scatter plot representation of all first four principal components combinations. (B) Barplot representation of the variance explained by each principal component. [file Image_1.TIFF]

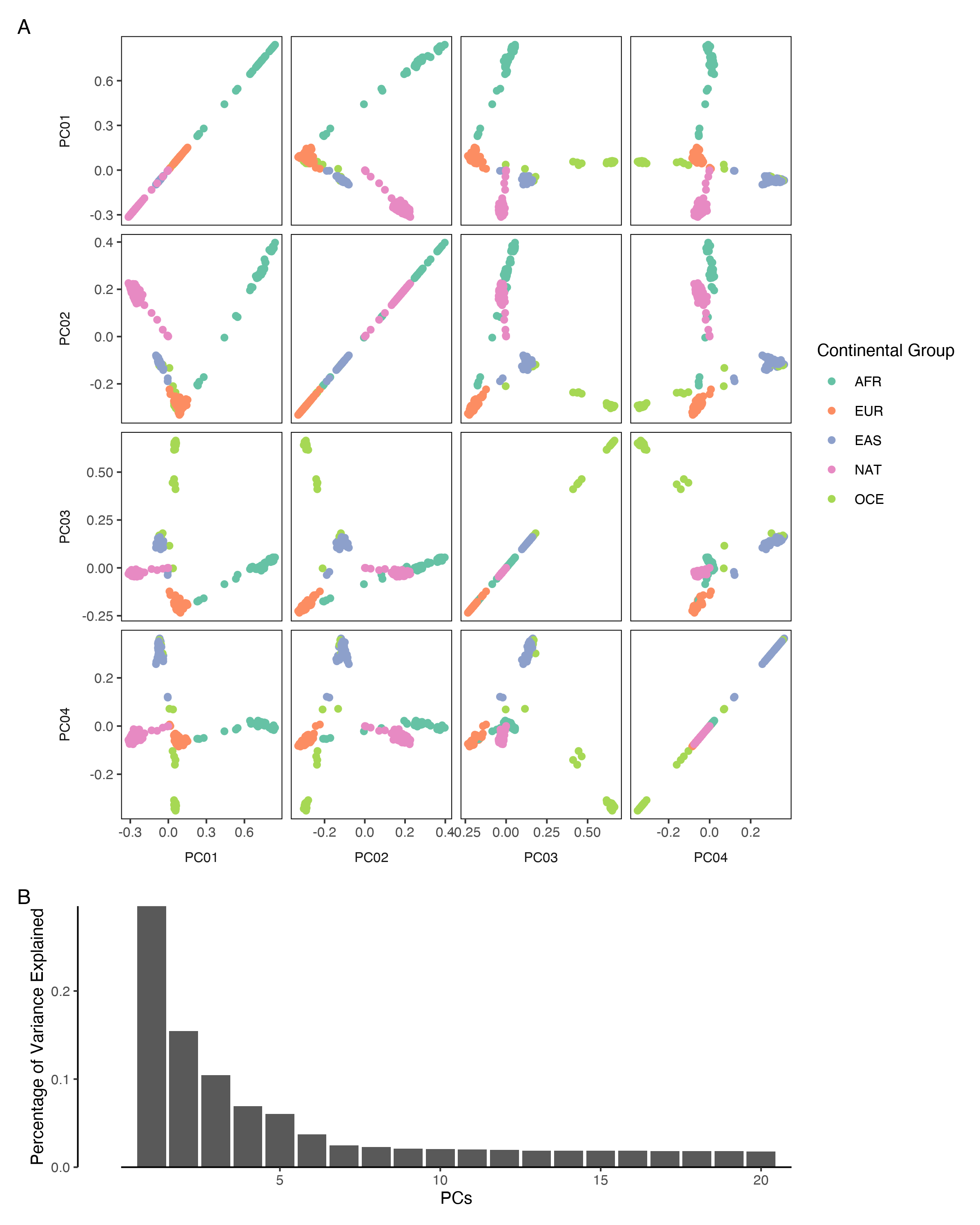

Supplement: Supplementary Figure 2 — PCA overview of all present-day worldwide sample analysis. Results of the PCA analysis conducted among all present- day worldwide samples included in this study. The samples were colored according to their geographic region with AFR indicating African populations, EAS East Asian, EUR European, OCE Oceanian and NAT Native American. (A) 2D Scatter plot representation of all first four principal components combinations. (B) Barplot representation of the variance explained by each principal component. [file Image_2.TIFF]

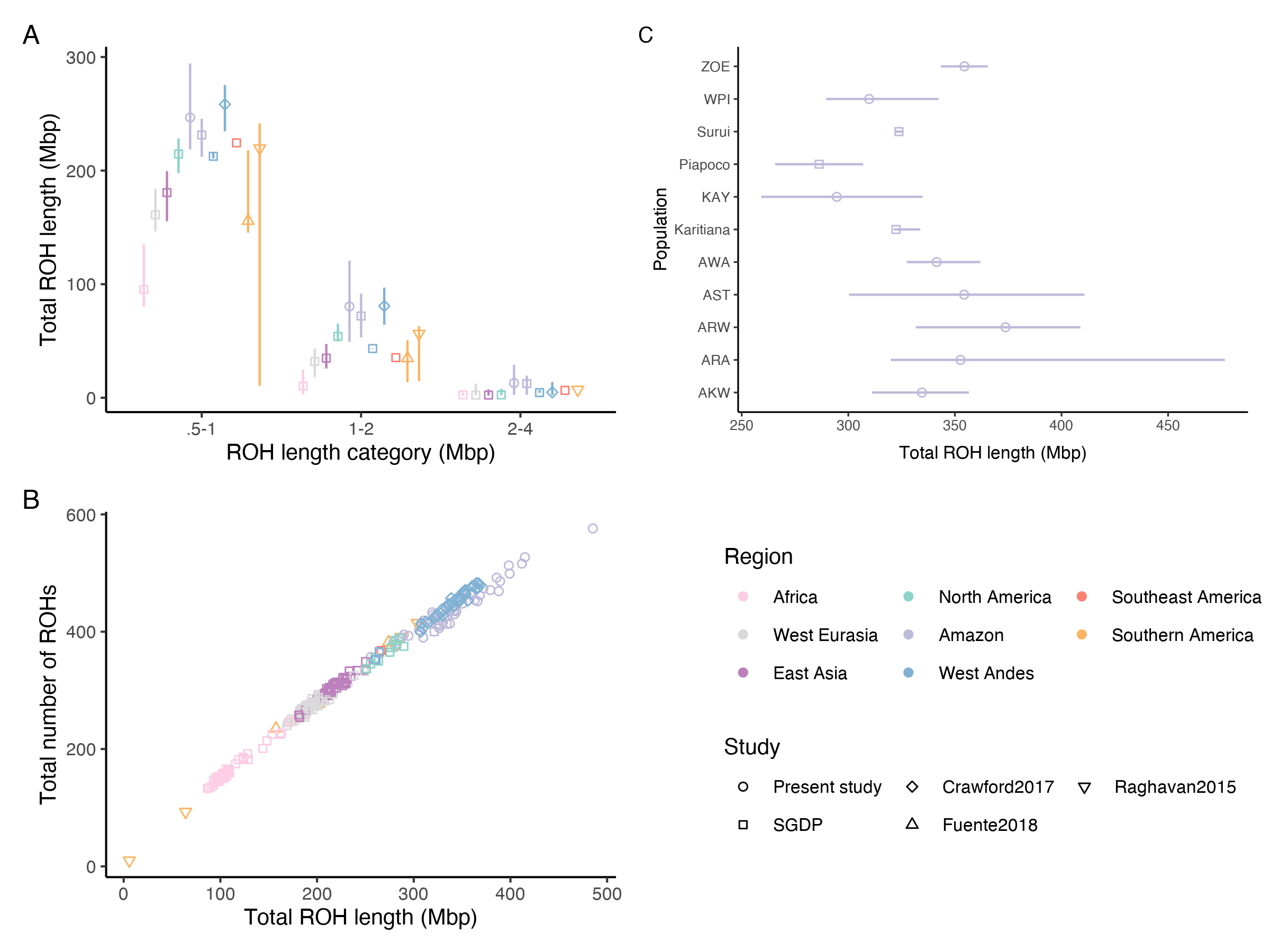

Supplement: Supplementary Figure 3 — Run of Homozygosity distribution in African, European, East Asian and Native American populations by study. The runs of homozygosity (ROH) were identified using plink v.1.9 and their distribution representation are colored according to the population geographic region and further distinguished by sample source study as indicated by the point shape. (A) Presents pointrange plots representing the median and 95% data interval of the total ROH length within each ROH length category. (B) Is a scatter plot showing the relation of total ROH length and the number of ROH of each individual. (C) Presents a pointrange plot representing the median and 95% data interval of total ROH length for each contemporan population included in the analysis. [file Image_3.TIFF]

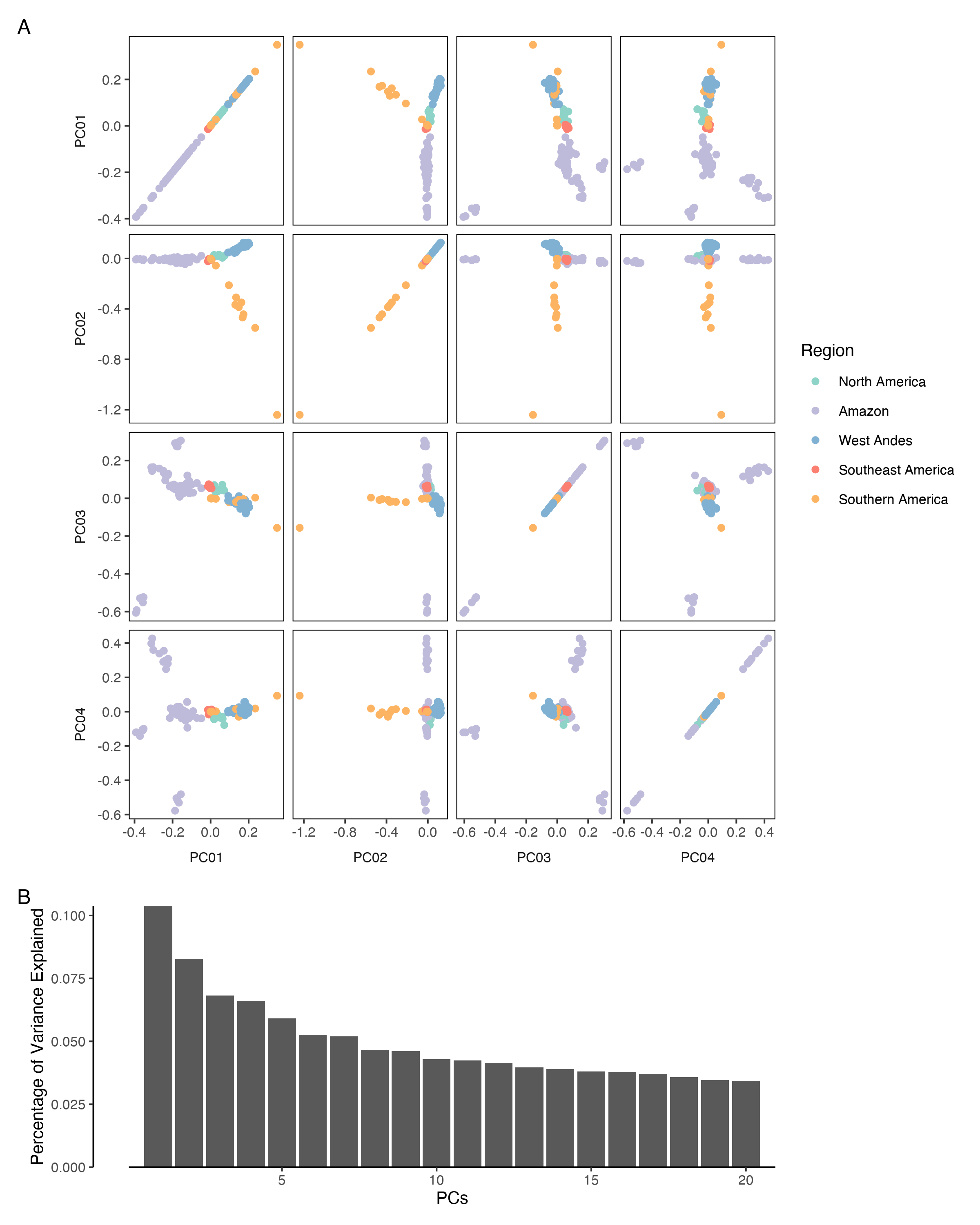

Supplement: Supplementary Figure 4 — PCA overview of all present-day Native American samples. Results of the PCA analysis conducted among all present-day native american samples included in this study. The samples were colored according to their geographic region, where North America was kept as is and South America was divided into: Amazon, West Andes, Southeast America and Southern America. (A) 2D Scatter plot representation of all first four principal components combinations. (B) Barplot representation of the variance explained by each principal component. [file Image_4.TIFF]

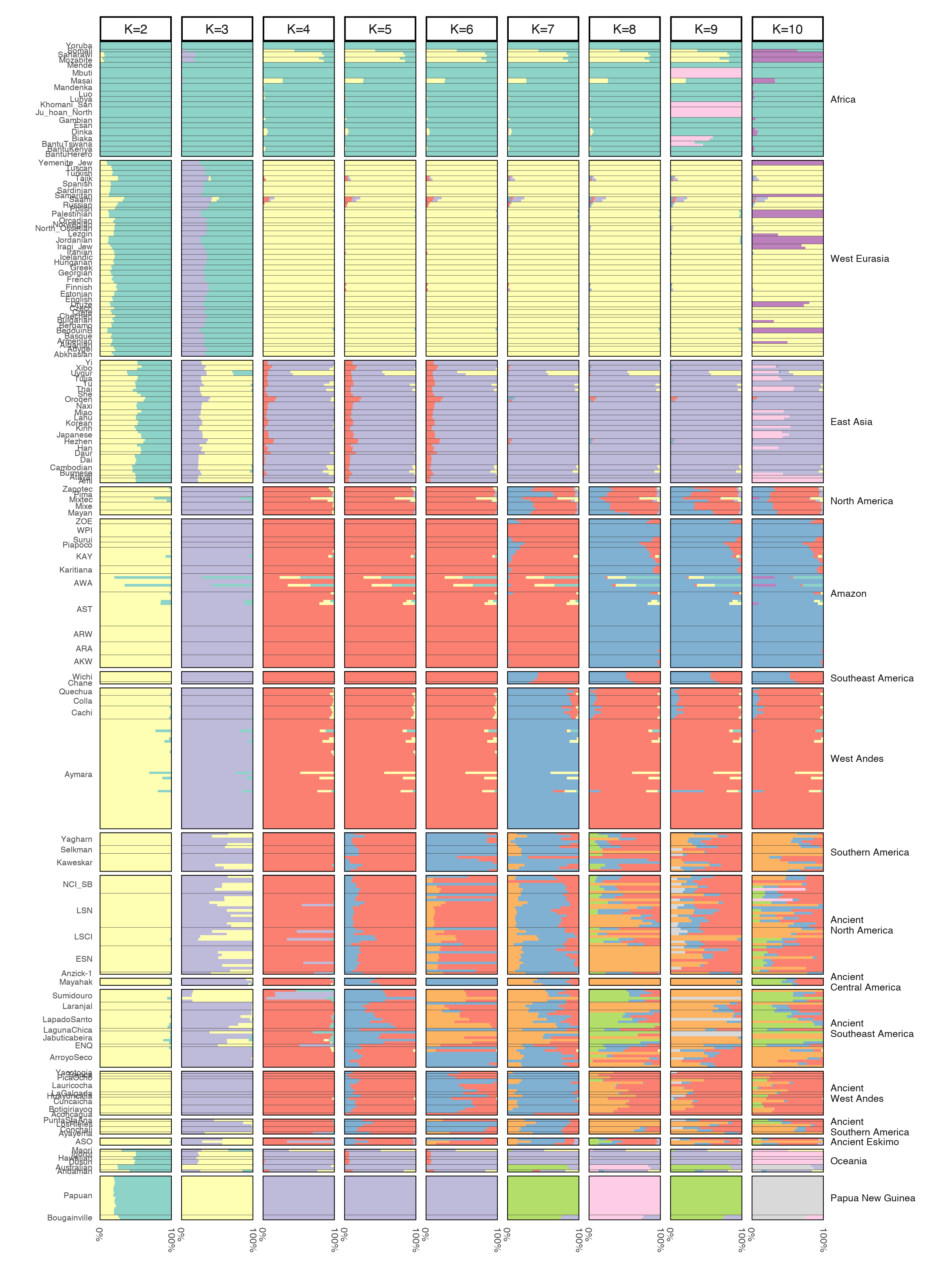

Supplement: Supplementary Figure 5 — Shared ancestry and genetic structure of the dataset. An unsupervised clustering analysis was performed with ADMIXTURE varying the number of putative ancestral components (K) from 2 to 10. Each horizontal bar represents an individual in the dataset and the colors represent their ancestry components assignments for each K putative ancestral. The samples were grouped according to their populations and geographic region which are indicated in y-axis and delimited by horizontal black bars and panel boxes, respectively. [file Image_5.TIFF]

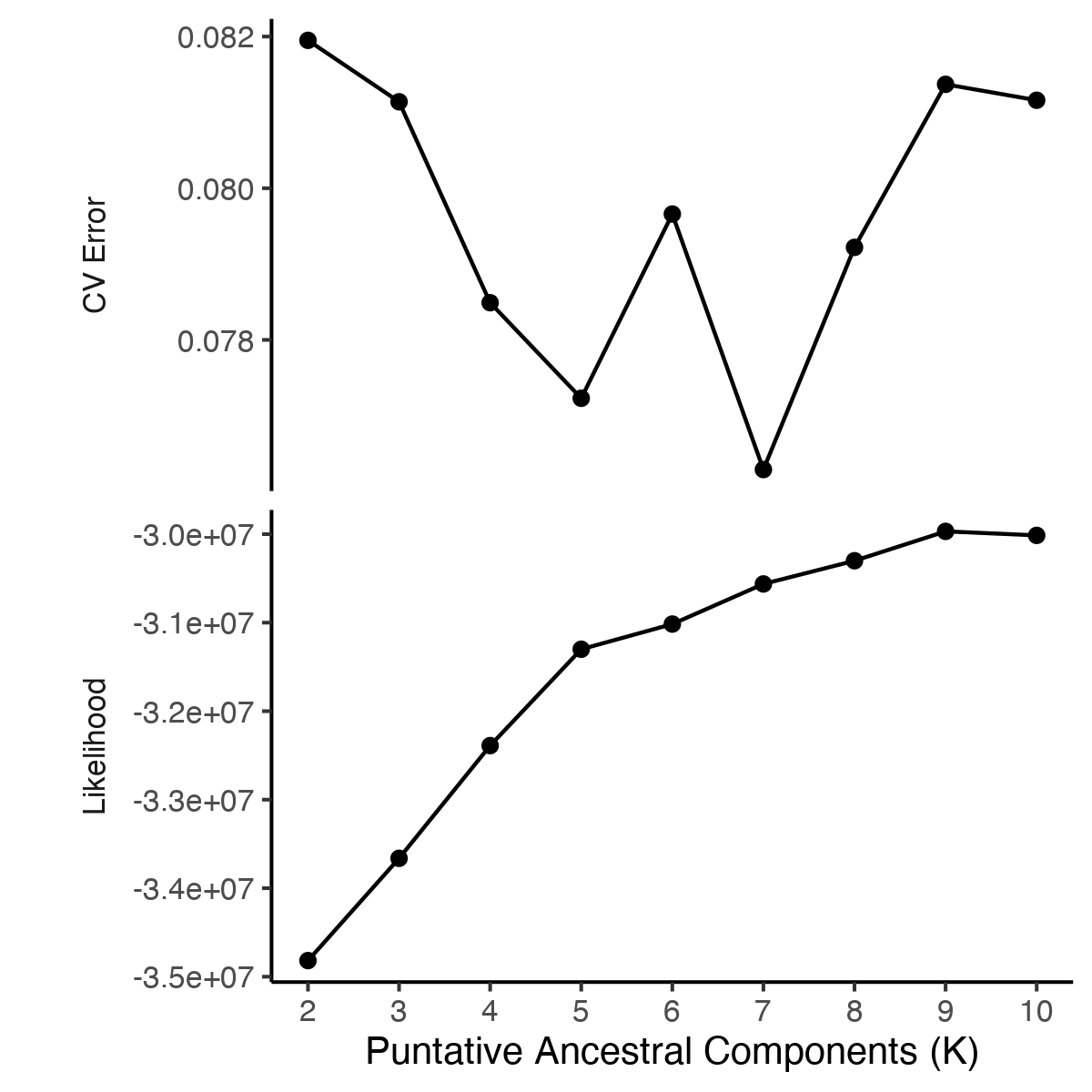

Supplement: Supplementary Figure 6 — ADMIXTURE genetic structure models likelihood and error. An unsupervised clustering was performed with ADMIXTURE to infer the contribution of putative ancestral components (K) varying from 2 to 10. Each model likelihood and cross-validation (CV) error were measured and presented above as a dot line plot. The measures indicate that the model with K = 7 presented the best fit to the data, considering it presented the lowest CV error. [file Image_6.TIFF]

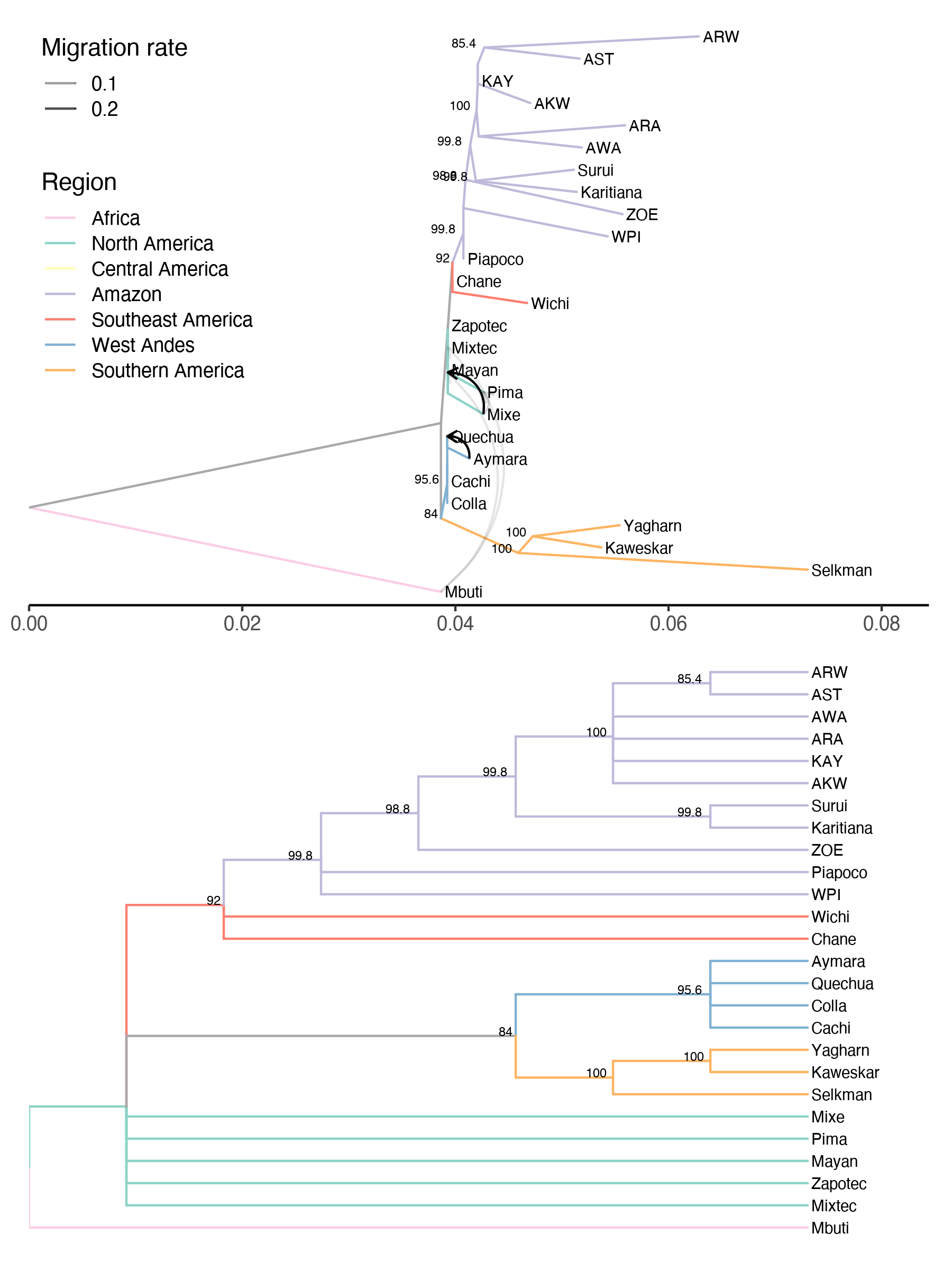

Supplement: Supplementary Figure 7 — Maximum Likelihood trees and admixture graph inferred. The maximum likelihood tree and admixture graphs were inferred with Treemix allowing up to five migration events between the branches. For this analysis the variants were blocked into 20,000 blocks and branch support were measured by 500 bootstrap iterations. The branches are colored according to the most recent common ancestor (MRCA) region and their support are indicated in both trees for those with over 75% support. Migration branches are indicated as curves and colored according to their migration ratio. In (A) we depict the maximum-likelihood tree produced by Treemix and in (B) we show the consensus tree produced by the bootstrap iterations where we merged all branches with less than 75% support. [file Image_7.TIFF]

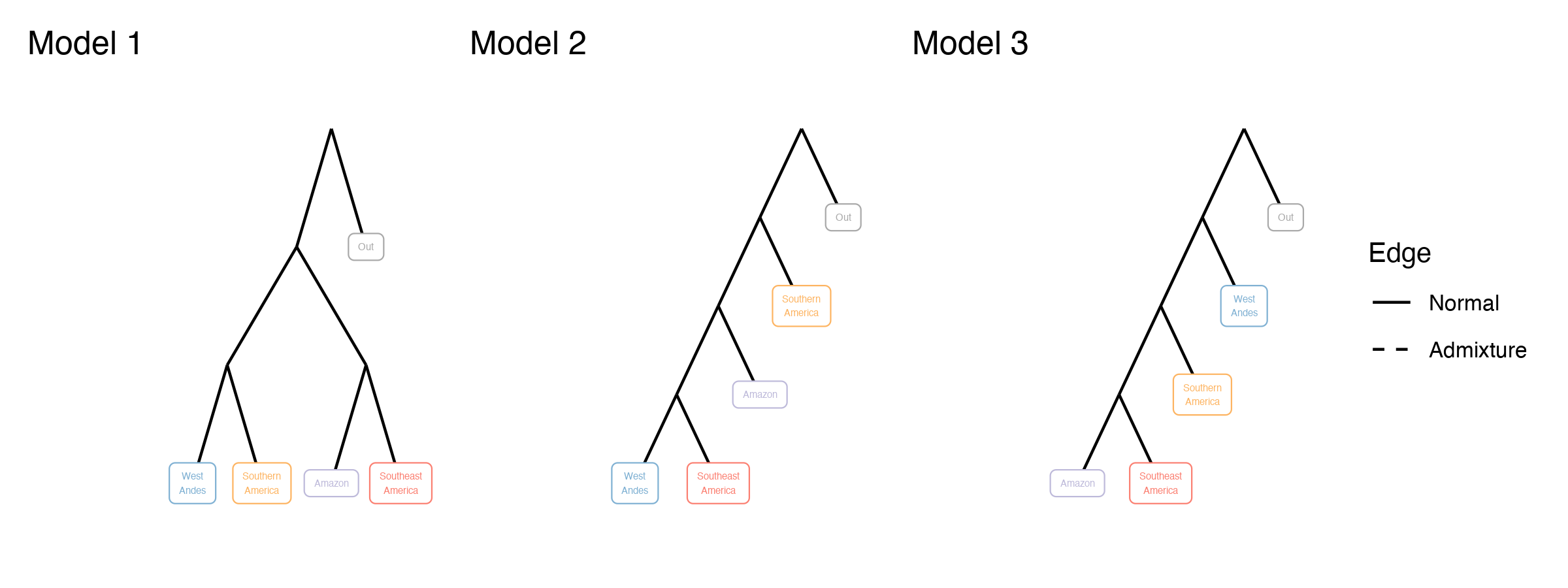

Supplement: Supplementary Figure 8 — Admixture graph representation of migration models proposed. To evaluate the migration models proposed in the manuscript, we’ve implemented them as admixture graphs to evaluate using qpGraph from ADMIXTOOLS. Admixture edges are represented as dashed lines and the four main South America regions and outgroup are indicated by their respective colors. Model 1 proposed that after being isolated from South Native American populations, West Andes and Amazon populations separated and the current South East is a product of their admixture. Model 2 instead proposed that Southeast America is a product of Southern America and Amazon admixture and Model 3 proposed that Southeast America shares a common ancestor to Amazon populations. [file Image_8.TIFF]

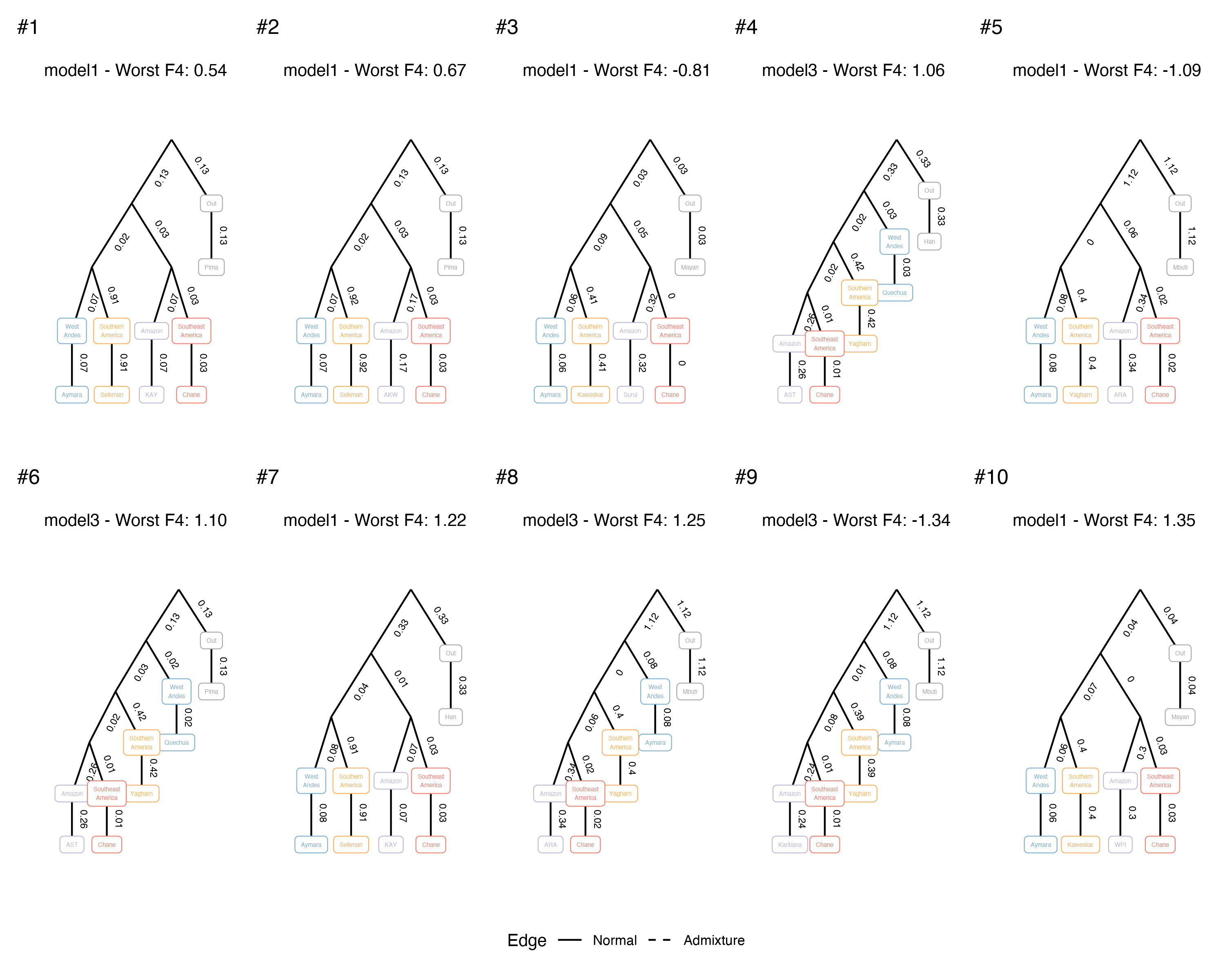

Supplement: Supplementary Figure 9 — Assessment of top ten fitted admixture graphs. The admixture graph for all three models were fitted using qpGraph from ADMIXTOOLS, varying the sample node representing each region. Here we present the top eight admixture graphs with the lowest likelihood. Admixture edges are represented by dashed lines with their contribution indicated as well as the edges Fst. [file Image_9.TIFF]

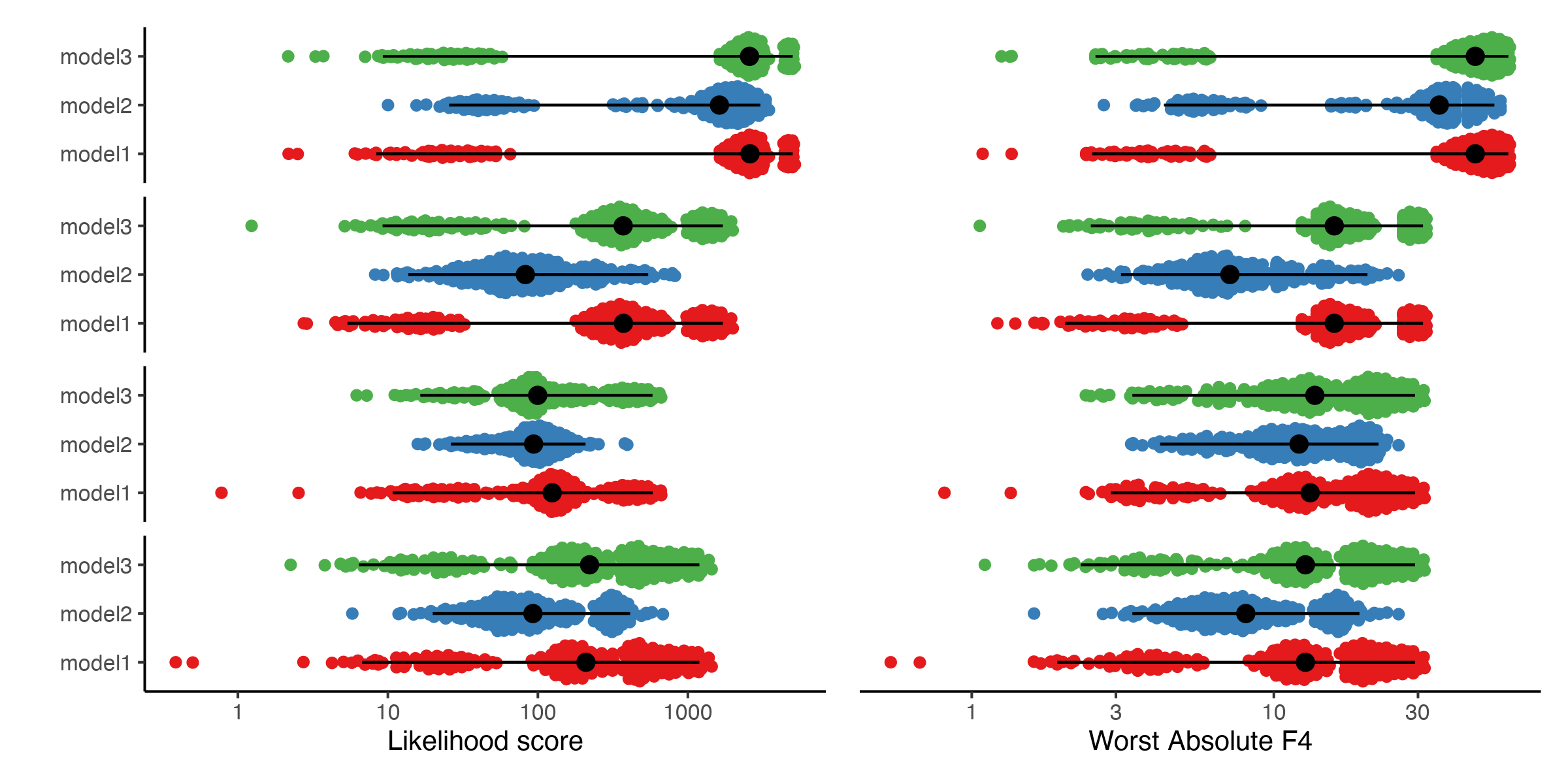

Supplement: Supplementary Figure 10 — Assessment of fitted admixture graphs. Each migration model proposed were evaluated with qpGraph function from the Admixtools2 package varying samples representing each leaf node (Out, Southern America, Amazon, West Andes, and Southeast America). Here we present a scatter plot of each model likelihood score (where smaller scores are better) and worst F4 obtained with either Mbuti, Han, Mayan, or Pima outgroups. Overall model 1 presents consistently better results than all others. The black pointrange indicates median and 95% data interval likelihood score and worst F4 for each model and outgroup. [file Image_10.TIFF]
