# Supplementary material for: Exome Sequencing of Native Populations From the Amazon Reveals Patterns on the Peopling of South America
Source: Front Genet. 2020 Oct 29;11:548507. doi: 10.3389/fgene.2020.548507 (PMC7660019; doi:10.3389/fgene.2020.548507)
Supplement: Supplementary Material 1 — Detailed Methods. [file Data_Sheet_1.PDF]

Supplementary Material for

Exome sequencing of Native Populations from the Amazon reveals patterns on the peopling of South America

André M. Ribeiro-dos-Santos[1], Amanda Ferreira Vidal[1], Tatiana Vinasco-Sandoval[1], João Guerreiro[1], Sidney Santos[1,2], Ândrea Ribeiro-dos-Santos[1,2] & Sandro J. de Souza[3,4,5*]

[1] Genetics and Molecular Biology Graduate Program, Instituto de Ciências Biológicas, UFPA, Belém, PA, Brazil

[2] Oncology and Medical Science Graduate Program, Núcleo de Pesquisas em Oncologica, UFPA, Belém, PA, Brazil

[3] Instituto do Cérebro, UFRN, Natal, Brasil.

[4] Bioinformatics Multidisciplinary Environment (BioME), Instituto Metrópole Digital, UFRN, Natal, Brasil.

[5] Institute of Systems Genetics, West China Hospital, Sichuan University, Chengdu, China.

*Corresponding author

Table of Contents

Detailed Methods 3

Sample collection and variant calling 3

Dataset Assembly 5

Genetic and Population Structure Analysis 5

References 9

Detailed Methods

Sample collection and variant calling

In the present study, we investigated exome variants of Native American populations within the Brazillian Amazon basin. A total of 58 blood samples were collected from eight Native American populations (***Figure 1***). Which included These included Araweté (ARW), Zo'é (ZOE), Wayãpy (WPI), Awa-Guajá (AWA) from the Tupi-Guarani language group; Asurini do Koatinemo (AKW), and Asurini do Trocará (AST) from the Asurini language group, which belong to the Tupi-Guarani language truck; Arara / Arara do Iriri (ARA) from the Karib language group; and Kayapó / Xikrin (KAY) from the Macro-Jê language group. In terms of geographical location, ARA, AKW and ARW are located on the Xingu River basin; KAY is located in the southeastern region of the Pará state; AST is located near the basin of the Tocantins river; ZOE inhabit a region between Cuminapanema and Erepecuru rivers in the northwestern portion of the Pará state; WPI inhabits the Oiapoque region and represents furthest north representant of the Tupi-Guarani language truck; and AWA is the last nomadic population in Brazil, they inhabit a vast region of the Maranhão state.

The genetic material was extracted from the whole blood samples using a phenol-chloroform method, quantified using Nanodrop fluorometer and evaluated integrity in 2% agarose gel electrophoresis. Next, whole exome sequencing libraries were prepared using Nextera Rapid Capture Exome (Illumina) and SureSelect Human XT All Exon V6 (Agilent) library preparation kits following manufacturer recommendations. The resulting libraries were sequenced in a NextSeq 550 platform (Illumina) in 4 NextSeq 500/550 High Output Kit runs with about 16 samples each, resulting in 43.6 Million read pairs per sample on average.

The reads were processed in our local server with Nextflow [1] workflow manager (available at https://www.nextflow.io/) using an analysis pipeline developed in our lab based on GATK Best Practices [2] and available at https://github.com/andremrsantos/exomeseq-nf. Briefly, poor quality reads were removed and trimmed using Trimmomatic v.0.36 [3] requiring a minimum length of 36 bases, trimming leading and trailing low quality bases with qual below 10 and cutting the reads when a 5 base sliding window average quality dropped below 15. The filtered reads were mapped to GRCh37 reference genome (https://www.ncbi.nlm.nih.gov/assembly/GCF_000001405.13) using BWA MEM v.0.7 [4] with the

default parameters and duplicates were marked and removed using Samblaster v.0.1 [5]. The mapping files were sorted and indexed using both Samtools v.1.8 [6] and Sambamba v.0.6 [7]. Finally, the mapped bases quality score were recalibrated based on various covariates at known variable sites using GATK v.4.0.0 (available at https://gatk.broadinstitute.org/) *BaseRecalibrator* and *ApplyBQSR* walkers.

The workflow yield on average 36.3 Million reads uniquely mapped to the genome (roughly 85% of all mapped reads) and 46.8X coverage of the exome targeted regions with 89% and 77% these bases with at least 10X and 20X of coverage, respectively. ***Supplementary Table 3*** includes a detailed table of these metrics for each sample.

Two strategies were applied for variant calling, an unguided and a guided approach. The unguided approach aimed to identify potential new variants and is consistent with the GATK best practices as described by Van der Auwera et al. [2]. Briefly, the variants within the exome target regions were identified using GATK v.4.0.0 HaplotypeCaller walker to produce a GVCF (Genomic Variant Call Format) for each sample which were aggregated into a single file using CombineGVCFs and finally the variants were genotyped as cohort call to improve precision using GenotypeGVCF. The variants quality were calculated using *VariantRecalibrator* and *ApplyVQSR* based on GATK resource bundle standard dataset (available at https://gatk.broadinstitute.org/hc/en-us/articles/360035890811-Resource-bundle), which included reference variant datasets for 1000 Genomes Project [8], HapMap [9]. SNV under 99.5% and INDELs under 99.0% sensitivity threshold were excluded. The variants were identified according to dbSNP 150 and annotated using SnpEff v.4.3 [10] and vcfanno v.0.3 [11] to include variants transcription effects, clinical importance according to the ClinVar[12], GWAS annotations from the GWAS catalog [13] and allele frequencies from ExAC, gnomAD [14] and 1000 Genomes Project [8]. The detailed code of these procedures is also included in the workflow available at *https://github.com/andremrsantos/exomeseq-nf*.

The guided approach aimed to maximize the number of comparable variants when realizing our population genetic analysis. We selected to analyze all variants of the Simons Genome Diversity Project Dataset (SGDP) [15] within the union of all target regions to genotype in our samples using GATK v.4.0.0 *HaplotypeCaller* using `--genotyping-mode GENOTYPE_GIVEN_ALLELES` mode and `--alleles` flag. Most external datasets investigated here were also genotyped using the procedure above, except for the SGDP which was the basis of this analysis and Pagani et al. [16] that didn't have raw sequencing data available.

Dataset Assembly

In this study, we've included public genetic data of present-day worldwide human populations from Simons Genome Diversity Project [15], Raghavan et al. [17], Crawford et al. [18], Pagani et al. [16], Fuente et al. [19], and of ancient samples from Rasmussen et al. [20], Scheib et al. [21] , Moreno-Mayar et al. [22] and Posth et al. [23] datasets. SGDP samples from Central Asia, Siberia and Southwest Asia were not included in this study. ***Supplementary Table 2*** includes the complete list of all samples included in this study.

The individual VCF (Variant Call Format) calls were aggregated into a single file and converted into a plink BED file using plink v.1.9 [24] excluding variants with missing genotype rate above 25%. Using this raw dataset, our samples relatedness was also inferred using KING v.2.1.4 [25] and full siblings samples were excluded from further analysis. Next, the ancestry proportions of putative ancestral components (i.e. African, European, East Asian, and Native American) were estimated by unsupervised clustering analysis with ADMIXTURE v.1.3.0 [26] with varying putative ancestral components (K) from 2 to 10 and evaluated by 5-fold cross-validation (***Supplementary Figures 5-6***).

On the analysis with five putative ancestral components (K=5), those components were classified as African, European, East Asian, Native American and Ancient Native American, according to the populations the components were predominant (green, yellow, purple, red, and blue ancestral components, respectively, on ***Supplementary Figure 5***). Native American samples with less than 90% contribution of either Native American or Ancient Native American were considered admixed and excluded from further analysis.

Genetic and Population Structure Analysis

The code used to conduct all analysis thereforward and generate all figures in the manuscript is available at the companion code repository at https://github.com/andremrsantos/paper-sa-population.

We broadly investigated the genetic structure and putative ancestral components contribution using the unsupervised ADMIXTURE v.1.3.0 [26] results mentioned above and principal component analysis conducted using flashPCA v.2.0 [27]. For the principal component analysis, flashPCA was run using default parameters including only variants with allele frequency above 1% for three sample subsets of the final dataset: (i) only Native American included in this study;

(ii) all contemporan Native American; and (iii) all contemporan samples (see ***Figure 1B, 2C,*** and ***Supplementary Figure 1-2, and 4***).

Based on the unsupervised ADMIXTURE models cross-validation error estimates, both models with 5 or 7 putative ancestral components presented the lowest error and high likelihood (***Supplementary Figure 6***). With the model of five putative ancestral components (K=5), the Native American samples are mostly composed by two putative ancestors denominated here as Native American and Ancient Native American (indicated in red and blue, respectively, in the ***Supplementary Figure 5***). While with the model of seven ancestral components (K=7), the Native American samples are mostly composed by three ancestral components denominated here as Amazonian, West Andean and Ancestral Native American (indicated in red, blue and orange, respectively, in the ***Supplementary Figure5***).

In order to further explore demographic patterns of the samples sequenced here, we conducted runs of homozygosity (ROH) pattern analysis and compared it to other the public samples investigated. ROH reflects demographic events since it is a product of the effective population size. Usually longer runs are the result of a recent event that limited the population genetic pool, such as inbreeding, bottlenecks and founder effects, whereas shorter ROHs indicated larger effective population size and points to much older events [28,29]. When considering the out-of-Africa model of human origins and dispersal, ROH presents a pattern of increment in respect to the walking distance from Africa and consequently a decrease of genetic diversity [28,29].

ROHs in our dataset were identified using PLINK v.1.9[24] requiring a length of at least 500kbp, at most 100kbp gap between SNPs, and at least one SNP per 50kbp, within a 50 SNPs sliding window used to extend the runs, a maximum of 1 heterozygous site and 25 missing calls were allowed, and at least 5% of windows need to overlap in order to form a segment (see `run-ROH.R` script at the companion repository for detailed steps). As shown in ***Supplementary Figures 3***, our dataset ROH patterns similar to the expected as described in the literature [28,29] with African populations presenting overall shorter runs, followed by European, East Asian, and Native American populations with the most and longest ROHs. The figures also show that samples sequenced here presented similar patterns to previously published samples from the Amazon and other Native South American populations.

Next, we estimated a maximum likelihood (ML) tree for all contemporan Native American samples in our dataset using the Mbuti population as an outgroup (***Supplementary Figure 7A***). The ML tree was estimated using Treemix v.1.13 [30] allowing to fit up to five migration edges

between the branches to improve the model fit. All variants with allele frequency above 1% among the analyzed samples were included and blocked into approximately 20,000 blocks. The branches confidence was estimated by 500 bootstrap iterations. Based on these iterations we have also estimated a consensus tree (***Supplementary Figure 7B***) by merging branches with less than 75% of support. Both ML and consensus trees indicate a clear separation of West Andes with Southern America populations and Amazonian with Southeast America (***Supplementary Figure 7***).

To measure gene-flow between the populations, f_3 and *D-statistics* were computed according to their descriptions by Reich et al. [31] and Patterson et al. [32], respectively, to evaluate our gene-flow models among Native South American populations. These statistics standard error and z-score were estimated by a weighted block jackknife approach as suggested by Reich et al. [31] and Patterson et al. [32] and similar to the one implemented in TreeMix v.1.13 [30]. This procedure consisted in measuring those statistics within a genomic block of approximately 100 variants and using these to compute average statistic, standard error and z-score, weighting the blocks' importance by the total number of non-missing sites within. These statistics were implemented as c++ code and made available in the companion code repository for this manuscript at https://github.com/andremrsantos/paper-sa-population.

Using these statistics we explore the controversial evidence of an Austrolasian genetic signal in Native Amazonian [22,23]. To test for such gene flow we computed D-statistics as ***D(Mbuti, Australasia; Mixe, X)*** for all 14 Amazonian populations as X included in the study and any of the following populations as Australasian: Andaman, Australian, Papuan New Guinea, Bougainville, Dusun, Igorot, and Maori (***Figure 3***). In such tests, z-values significantly greater than 0 indicate an Australasian genetic signal.

We hypothesize that the occupation of South America started from at least two distinct coastal routes, one Pacific and one Atlantic. We tested different demographic models using the following outgroup D-statistics as ***D(Mbuti, X; Y, Z)*** with Mbuti as outgroup, X as the test population, Y and Z are the populations for which we are comparing the genetic drift (***Figure 4***). Z-values significantly higher than 0 indicate a closer proximity of test population X to Z and significantly lower than 0 indicate a closer proximity to Y. We tested the following three formats:

- ***D(Mbuti, X; Amazon, Southern America)***, which measures if the test population X genetic drift in regards to Amazonian and Southern America populations. Where Z-value

significatively higher than 0 indicate closer proximity to Southern America and lower than 0 indicate closer proximity to Amazon populations.

- ***D(Mbuti, X; Amazon, West Andes)***, which measures if the test population X genetic drift in regards to Amazonian and West Andean populations.
- ***D(Mbuti, X; Southern America, West Andes)***, which measures if the test population X genetic drift in regards to Southern America and West Andean populations.

Figure 4 showed that all populations sequenced here show a clear and distinct Amazonian genetic signal and support the PCA, and ADMIXTURE results of the separation between western and eastern Native American populations.

Based on these results and TreeMix ML tree, we envisioned four potential scenarios for the occupation of South America: 1) groups from the Atlantic route eventually turned south to occupy the Amazon basin and the Southeast of the continent; 2) groups from the Pacific route eventually turned east and occupied the central parts of the continent (likely in different waves from north to south); 3) Andean groups in the south migrate east and eventually turned north to occupy the south/central part of Brazil and the Amazonian region; and 4) a mixed model involving at least two of the models above. Since scenario 4 above is difficult to test with the present limitation in sample size, this leaves us with scenarios 1, 2 and 3.

We modeled these scenarios as admixture graphs (***Supplementary Figure 8***) and computed the models likelihood and worst f_4 statistic using qpGraph from admixtools v.2.0 (available at https://uqrmaie1.github.io/admixtools/index.html) while varying the population representing each leaf nodes. Overall model 1 better fitted the data with smaller likelihood scores and f_4 statistics with few tests presenting the worst absolute f_4 below 3 (***Supplementary Figure 9-10***). Even Though model 2 presented a median score lower than model 1 and 3, model 2 minimal score was far greater than observed in those models and none test format presented the worst absolute f_4 below 3.

References

1. Di Tommaso, P. *et al.* Nextflow enables reproducible computational workflows. *Nat. Biotechnol.* **35**, 316–319 (2017).
2. Van der Auwera, G. A. *et al.* From FastQ data to high confidence variant calls: the Genome Analysis Toolkit best practices pipeline. *Curr. Protoc. Bioinforma.* **43**, 11.10.1-11.10.33 (2013).
3. Bolger, A. M., Lohse, M. & Usadel, B. Trimmomatic: a flexible trimmer for Illumina sequence data. *Bioinforma. Oxf. Engl.* **30**, 2114–2120 (2014).
4. Li, H. Aligning sequence reads, clone sequences and assembly contigs with BWA-MEM. *ArXiv13033997 Q-Bio* (2013).
5. Faust, G. G. & Hall, I. M. SAMBLASTER: fast duplicate marking and structural variant read extraction. *Bioinformatics* **30**, 2503–2505 (2014).
6. Li, H. *et al.* The Sequence Alignment/Map format and SAMtools. *Bioinforma. Oxf. Engl.* **25**, 2078–2079 (2009).
7. Tarasov, A., Vilella, A. J., Cuppen, E., Nijman, I. J. & Prins, P. Sambamba: fast processing of NGS alignment formats. *Bioinforma. Oxf. Engl.* **31**, 2032–2034 (2015).
8. 1000 Genomes Project Consortium *et al.* A global reference for human genetic variation. *Nature* **526**, 68–74 (2015).
9. Gibbs, R. A. *et al.* The International HapMap Project. *Nature* **426**, 789–796 (2003).
10. Cingolani, P. *et al.* A program for annotating and predicting the effects of single nucleotide polymorphisms, SnpEff: SNPs in the genome of Drosophila melanogaster strain w1118; iso-2; iso-3. *Fly (Austin)* **6**, 80–92 (2012).
11. Pedersen, B. S., Layer, R. M. & Quinlan, A. R. Vcfanno: fast, flexible annotation of genetic variants. *Genome Biol.* **17**, 118 (2016).
12. Landrum, M. J. *et al.* ClinVar: public archive of relationships among sequence variation

and human phenotype. *Nucleic Acids Res.* **42**, D980-985 (2014).

13. MacArthur, J. *et al.* The new NHGRI-EBI Catalog of published genome-wide association studies (GWAS Catalog). *Nucleic Acids Res.* **45**, D896–D901 (2017).

14. Lek, M. *et al.* Analysis of protein-coding genetic variation in 60,706 humans. *Nature* **536**, 285–291 (2016).

15. Mallick, S. *et al.* The Simons Genome Diversity Project: 300 genomes from 142 diverse populations. *Nature* **538**, 201–206 (2016).

16. Pagani, L. *et al.* Genomic analyses inform on migration events during the peopling of Eurasia. *Nature* **538**, 238–242 (2016).

17. Raghavan, M. *et al.* Genomic evidence for the Pleistocene and recent population history of Native Americans. *Science* **349**, (2015).

18. Crawford, J. E. *et al.* Natural Selection on Genes Related to Cardiovascular Health in High-Altitude Adapted Andeans. *Am. J. Hum. Genet.* **101**, 752–767 (2017).

19. Fuente, C. de la *et al.* Genomic insights into the origin and diversification of late maritime hunter-gatherers from the Chilean Patagonia. *Proc. Natl. Acad. Sci.* **115**, E4006–E4012 (2018).

20. Rasmussen, M. *et al.* The genome of a Late Pleistocene human from a Clovis burial site in western Montana. *Nature* **506**, 225–229 (2014).

21. Scheib, C. L. *et al.* Ancient human parallel lineages within North America contributed to a coastal expansion. *Science* **360**, 1024–1027 (2018).

22. Moreno-Mayar, J. V. *et al.* Early human dispersals within the Americas. *Science* **362**, (2018).

23. Posth, C. *et al.* Reconstructing the Deep Population History of Central and South America. *Cell* **175**, 1185-1197.e22 (2018).

24. Chang, C. C. *et al.* Second-generation PLINK: rising to the challenge of larger and richer datasets. *GigaScience* **4**, 7 (2015).

25. Manichaikul, A. *et al.* Robust relationship inference in genome-wide association studies. *Bioinformatics* **26**, 2867–2873 (2010).

26. Alexander, D. H. & Lange, K. Enhancements to the ADMIXTURE algorithm for individual ancestry estimation. *BMC Bioinformatics* **12**, 246 (2011).

27. Abraham, G., Qiu, Y. & Inouye, M. FlashPCA2: principal component analysis of Biobank-scale genotype datasets. *Bioinformatics* **33**, 2776–2778 (2017).

28. Kirin, M. *et al.* Genomic Runs of Homozygosity Record Population History and Consanguinity. *PLOS ONE* **5**, e13996 (2010).

29. Ceballos, F. C., Joshi, P. K., Clark, D. W., Ramsay, M. & Wilson, J. F. Runs of homozygosity: windows into population history and trait architecture. *Nat. Rev. Genet.* **19**, 220–234 (2018).

30. Pickrell, J. K. & Pritchard, J. K. Inference of Population Splits and Mixtures from Genome-Wide Allele Frequency Data. *PLOS Genet.* **8**, e1002967 (2012).

31. Reich, D., Thangaraj, K., Patterson, N., Price, A. L. & Singh, L. Reconstructing Indian Population History. *Nature* **461**, 489–494 (2009).

32. Patterson, N. *et al.* Ancient Admixture in Human History. *Genetics* **192**, 1065–1093 (2012).
